# *Acacia mangium*: A promising plant for isolating anti-hepatitis C virus agents

**DOI:** 10.12688/f1000research.124947.1

**Published:** 2022-12-08

**Authors:** Tutik Sri Wahyuni, Nida S. Sukma, Adita A. Permanasari, Chie Aoki-Utsubo, Aty Widyawaruyanti, Achmad Fuad Hafid

**Affiliations:** 1 Center of Natural Product Medicine Research and Development, Institute of Tropical Disease, Airlangga University, Surabaya, Indonesia; 2 Department Pharmaceutical Science, Faculty of Pharmacy, Airlangga University, Surabaya, Indonesia; 3 Undergraduate student, Faculty of Pharmacy, Airlangga University, Surabaya, Indonesia; 4 Dept. of Public Health, Kobe University Graduate School of Health Sciences, Kobe, Japan

**Keywords:** Acacia mangium, hepatitis C virus, infectious disease, medicinal plant, medicine, health

## Abstract

**Background:** Medicinal plants are potential resources for isolating drug candidates. Various plants have been reported to possess pharmacological effects including anti-hepatitis C activities. The current study examined the anti-hepatitis C virus (HCV) activities of *Acacia mangium* extracts in solvents with various polarities and further evaluated the mechanism of action of the extracts using Western blotting and combination treatment models.

**Methods:** The leaves of *A. mangium* were extracted in two phases, first in ethanol and then in solvents with different polarities (n-hexane, dichloromethane, and methanol). HCV-infected Huh7it-1 cells were treated with the extracts at concentrations of 0.01, 0.1, 1, 10, 50, and 100 µg/mL.

**Results:** The results revealed the strong anti-HCV activities of the extracts. The 50% inhibition concentrations (IC _50_s) of the ethanol, n-hexane, dichloromethane and methanol extracts were of 4.6 ± 0.3, 2.9 ± 0.2, 0.2 ± 0.3, and 2.8 ± 0.2 μg/mL, respectively, and no cytotoxic effect was detected. These extracts displayed stronger effects than the positive control ribavirin. The mode of action of the ethanol extract was evaluated at 30 µg/mL, revealing that the inhibitory effect was stronger on the post-entry step than on the entry step. Western blotting revealed that the extracts decreased NS3 protein expression, indicating that virus replication was suppressed. Further evaluation illustrated that combined treatment with the ethanol extract enhanced the anti-viral activity of simeprevir.

**Conclusions:** These results indicated that *A. mangium* leaves could represent sources of anti-HCV agents.

## Introduction

Hepatitis C virus (HCV) infection is an acute or chronic liver disease. HCV infection has a high prevalence globally, and approximately 71 million people are at risk of liver cirrhosis or hepatocellular carcinoma attributable to chronic infection ( Lange *et al.*, 2014). To date, no effective hepatitis C vaccine has been developed because HCV is a commonly mutated virus ( Pawlotsky *et al.*, 2015; Pawlotsky Jm et al., 2018).

Hepatitis C treatment has evolved with the availability of direct acting antivirals, which have achieved sustained virological response (SVR) rates exceeding 90% ( Javed *et al.*, 2011). However, some low-income countries cannot access those therapies because of their costs, and the combination of interferon-alpha and ribavirin (RBV), which produces an SVR rate of 50%, remains in use. Combination treatment has also been reported to have serious side effects and risks of resistance, making this strategy less effective ( Swain *et al.*, 2010). Efforts to develop new agents for HCV are necessary. Further issues to overcome include the development of drugs that can inhibit the virus with fewer side effects and affordable prices for all countries. Therefore, it is necessary to develop affordable, safe, and effective HCV therapies.

Traditional herbal medicine has become a popular treatment, and plants are among the primary components of such medicines. Our previous studies reported medicinal plants possessing anti-HCV activities ( Adianti *et al.*, 2014; Wahyuni *et al.*, 2013; Wahyuni *et al.*, 2014). Many medicinal plants have also been reported to inhibit HCV by inhibiting various steps of the HCV life cycle ( Hussein *et al.*, 2000; Ravikumar *et al.*, 2011; Wahyuni *et al.*, 2016).

Plants in the Fabaceae family are frequently used by traditional healers to treat liver diseases, including HCV infection. The chemical compounds present in the Fabaceae family include saponins, tannins, flavonoids, proteins, stylbenoid, xanthones, terpenes (triterpenes, diterpenes), phytoalexin, galactonate, lactogenic agents (polyketide), and anthraquinone. Most of those compounds are reported to possess anti-viral, hepatoprotective, and anti-cancer activities ( Roy *et al.*, 2016). One genus of the Fabaceae family that has been demonstrated to inhibit HIV is *Acacia*, and the active species include *A. nilotica* (50% inhibitory concentration [IC _50_] = 40.5 μg/mL) and *A. confusa* (IC _50_ = 5 μg/mL) ( Hussein *et al.*, 2000; Lee *et al.*, 2011).

*A. mangium* contains alkaloids, flavonoids, polyphenols, glycosides, saponins, steroids, tannins, and terpenoids, and their leaves contain phenolic groups including tannins and flavonoids. *A. mangium* was reported to contain 2,3-cis-3,4,7,8-tetrahydroxyflavanone and teracacidin ( Barry *et al.*, 2005). This current study evaluated the anti-HCV activity of various extracts of *A. mangium.* In addition, the cytotoxicity of the extracts was evaluated. The mode of action was additionally assayed to determine the part(s) of the HCV life cycle inhibited by the extracts. Moreover, its mechanism of action was examined by Western blotting and combination treatment with current anti-HCV drug.

## Methods

### Materials

*A. mangium* leaves were obtained from an area in Mojokerto Regency, Indonesia. The plant was verified by an expert botanist from Materia Medica Indonesia, East Java (see the *Underlying data* ( Wahyuni, 2022)).

The materials used in the bioassays were as follows: Huh7it-1 cells ( Apriyanto *et al.*, 2016); adapted variant of HCV strain (JHF1a) ( Yu *et al.*, 2010); Dulbecco’s Modified Eagle’s Medium (DMEM, GIBCO Invitrogen) supplemented with 10% of fetal bovine serum (FBS, GIBCO Invitrogen), 150 μg/mL kanamycin (Sigma-Aldrich), and non-essential amino acids (NEAAs, GIBCO Invitrogen); Dulbecco’s phosphate-buffered saline (GIBCO Invitrogen); formaldehyde (HCHO, Sigma-Aldrich); trypsin-EDTA (Sigma-Aldrich); 3-(4,5-dimethylthiazol-2-yl)-5-(3-carboxymethoxyphenyl)-2-(4-sulfophenyl)-2H-tetrazolium (MTT, Sigma-Aldrich); bovine serum albumin (BSA, Biowest); Triton X-100 Sigma-Aldrich); 3,3′-diaminobenzidine (DAB, Thermo Fisher Scientific); anti-HCV human antibody and HRP-conjugated goat anti-human Ig antibody (Thermo Fisher Scientific); RIPA buffer; polyacrylamide gel and polyvinylidene difluoride (PVDF) membranes (Millipore, Bedford, MA, USA); β-actin antibody (MBL, Nagoya, Japan); and a chemiluminescence detection system (Bio-Rad; GE Healthcare, UK).

### Preparation of *A. mangium* leaf extracts

*A. mangium* leaves (2 kg) were dried, ground into powder, and further extracted with two kind of extraction procesess. First, 200 g of powder was extracted by maceration process with a total of 2 liters of 96% ethanol and another 200 g was successively extracted with 2 liters of n-hexane, dichloromethane, and methanol. Specifically, 10 mg of the extract powder were dissolved in 100 μL of dimethyl sulfoxide to obtain 100,000 μg/mL stock solution ( Wahyuni *et al.*, 2013).

### Cell and virus culture

Huh7it-1 cells were cultured in DMEM supplemented with 10% FBS, 150 μg/mL kanamycin, and NEAAs in 5% CO _2_ at 37°C and maintained for bioassay purposes. Cells were incubated at 37 °C for 2 days. Cells which showed more than 80% confluence were used for further bioactivity assay. The detailed protocol for cell passage is available at https://dx.doi.org/10.17504/protocols.io.n92ldpbd7l5b/v1.

Virus stock was obtained by propagating HCV in Huh7it-1 cells. Culture supernatants at day 3, 5 and 7 after virus infection were collected. Virus titers were calculated by titration assay (wahyuni, 2013). In brief the virus harvested was diluted on x5, x25, x125, x625, and x3125 then put onto Huh7it-1 cells and incubated for 4 hours, the remaining virus was removed and refed with new medium for further incubation for 2 days. The infected cells were stained with DAB staining reagent and further calculated. The number of viruses represented as titer virus. The high titer virus stock (higher than 1x10 ^5^) was chosen for anti-HCV assay. The stock was stored at −80°C until use ( Wahyuni *et al.*, 2018).

### Anti-HCV activity

An anti-HCV assay was conducted using HCV-infected Huh7it-1 cells. Various concentrations of the extracts were mixed with virus solution (multiplicity of infection of 0.1) and inoculated into the cells at a final concentration of 0.01, 0.1, 1, 10, 50, or 100 μg/mL. The cultures were incubated for 48 h with 2 h of virus inoculation and further incubation for 46 hours at 37°C. The viral levels of the supernatants were examined by titration assay. Culture cell supernatant was collected, diluted 10x with medium and inoculated to the Huh7it cells. This was incubated for 2 days and the infected cells were calculated after the immunostaining process. The inhibitory effect of the extracts were calculated compared to the untreated control. The 50% inhibitory activity was conducted by SPSS software version 25 ( Wahyuni *et al.*, 2019; Wahyuni *et al.*, 2018). Ribavirin was used as the positive control.

### Viral titration and immunostaining

Huh7it-1 cells were incubated with serial dilutions of the supernatant for 48 h. The cells were fixed with formaldehyde, stabilized with triton and subjected to immunostaining with primary antibody (human serum) and secondary antibodies (HRP-conjugated goat anti-human). The detailed immunostaining protocol is available in the *Underlying data* ( Permanasari and Wahyuni, 2022a). DAB staining was performed to visualize the infected cells. The percent inhibitory effect was calculated by comparing the reduction of infected cells to the control ( Wahyuni *et al.*, 2018).

### Mode of action analysis

The mode of action assay was performed to examine whether *A. mangium* extract affected the entry or post-entry step of the HCV life cycle. Three parallel experiments were performed. First, the extract was only added to the cells during viral inoculation. Second, the extract was only added to the cells after viral inoculation. Third, the cells were treated with the extract both during and after inoculation. After 48 h of incubation, all supernatants were collected to examine their viral levels by titration assay. Viral supernatant was diluted with medium and inoculated to the Huh7it cells. Infected cells were calculated to further determine the percentage inhibitory against HCV. Anti-HCV activity was expressed by 50% inhibition concentration (IC _50_) ( Hafid *et al.*, 2017).

### Cytotoxicity assay

The MTT (3-(4,5- Dimethylthiazol-2-yl)-2,5-Diphenyltetrazolium Bromide) assay was used to measure cytotoxicity. Huh7it-1 cells were incubated with various concentrations of the extracts for 48 h. Then, 10% MTT was added to the cultures for 4 h. Absorbance was measured at 560 and 750 nm to calculate the percentage cell viability relative to the control. SPSS software version 25 probit analysis was used to calculate the 50% cytotoxic concentration (CC _50_) ( Wahyuni *et al.*, 2018). The protocol of the MTT assay is available at http://dx.doi.org/10.17504/protocols.io.6qpvr4x5pgmk/v1.

### Immunoblotting assay

Huh7it-1 cells were treated with mixtures of the extracts (10 or 50 μg/mL) and HCV. After incubation for 2 days at 37 °C, the cells were collected, lysed, and protein levels were determined using a BCA assay kit (Thermo Fisher Scientific). Equal amounts of proteins were subjected to SDS–polyacrylamide gel electrophoresis followed by transfer to a polyvinylidene difluoride membrane. Samples were run in transfer buffer at 0.3 A for 30 minutes followed by processing to SDS running buffer at 0.1 A for 25 minutes. The membrane was applied into a blocking buffer of skim milk and reacted to antibodies. The primary antibody was an HCV NS3 mouse monoclonal antibody (clone H23; Abcam, Cambridge, MA, USA), and the secondary antibody was HRP-conjugated goat anti-mouse immunoglobulin. β-actin (MBL, Nagoya, Japan) served as the internal control ( Permanasari *et al.*, 2021; Widyawaruyanti *et al.*, 2021). Membranes were incubated at room temperature for 1 hour in each antibody. NS3 protein expression was detected using an enhanced chemiluminescence detection system (GE Healthcare, Buckinghamshire).

### Anti-HCV activity of the combination of *A. mangium* extract and simeprevir

Combination treatment was performed by adding equal volumes of *A. mangium* extract and simeprevir (Toronto Research Chemical, Canada). Simeprevir was added at 0.25×, 0.5×, 1×, 2×, and 4×IC _50_ for monotherapy and combination. All treatments were performed for 48 h incubation. The IC _50_ of simeprevir when used in combination with *A. mangium* extract and monotherapy were calculated and compared using the SPPS probit assay version 25 ( Wahyuni et al., 2020).

## Results

### *In vitro* activity of *A. mangium* against HCV

**Figure 1.**
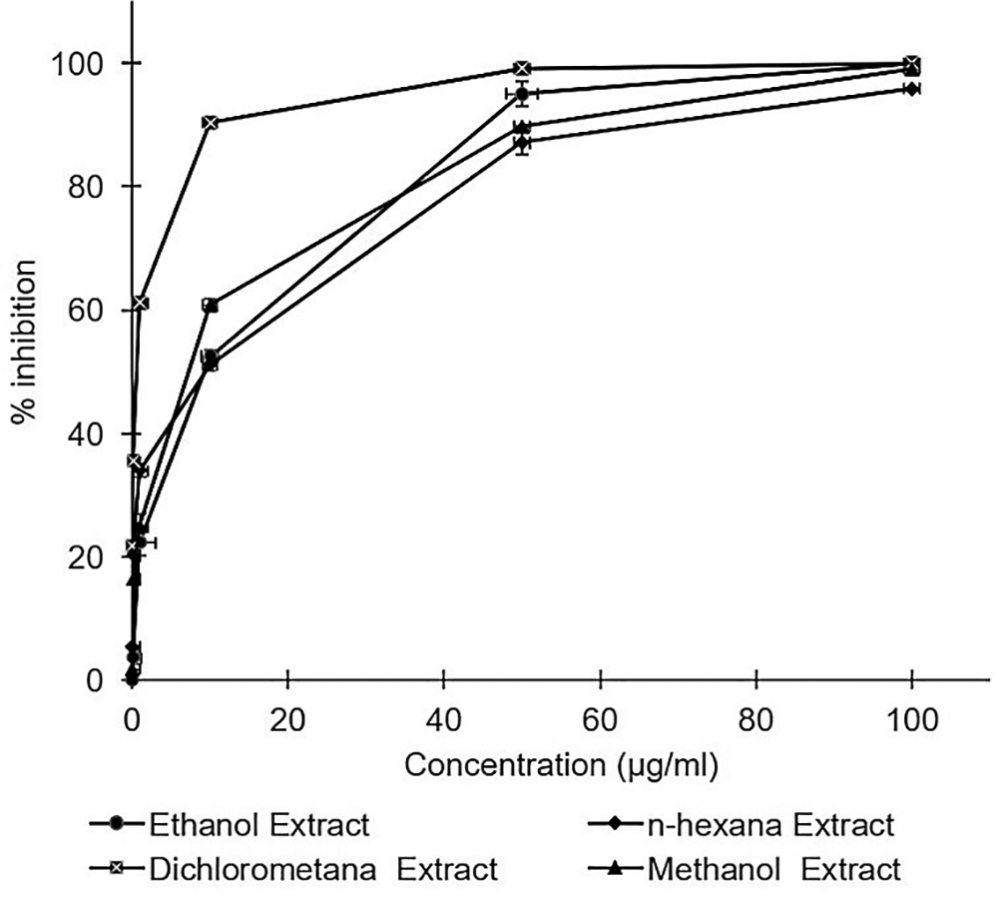
Concentration-dependent inhibition of hepatitis C virus infectivity by the ethanol, n-hexane, dichloromethane, and methanol extracts of *Acacia mangium.* Huh7it-1 cells were cultured and inoculated with mixtures of the virus and each extract at various concentrations. Virus inhibition was calculated relative to the untreated control. The data represent the mean of three independent experiments.

All *A. mangium* leaf extracts strongly inhibited HCV in a dose dependent manner ( Figure 1). Inhibition concentrations of 50% of all extracts were calculated by probit analysis. Dichloromethane extract displayed the strongest effects, with an IC _50_ of 0.2 ± 0.3 μg/mL, whereas the IC _50_s of the extracts ranged 2.8–4.6 μg/mL ( Table 1). While the positive control of ribavirin revealed the IC _50_ values of 10.4 ± 0.2 μg/mL. All of the extracts were demonstrated to possess stronger activity compared to the positive control of ribavirin. The raw data are available in the *Underlying data* ( Permanasari and Wahyuni, 2022b).

**Table 1.** The 50% inhibitory concentration (IC _50_) and 50% cytotoxic concentration (CC _50_) of *Acacia mangium* leaf extracts.

Extract/fraction	IC _50_ (μg/mL)	CC _50_ (μg/mL)	Selectivity index (CC _50_/IC _50_)
Ethanol 96%	4.6 ± 0.3	>400	>86.9
n-Hexane	2.9 ± 0.2	121.2 ± 1.2	41.8
Dichloromethane	0.2 ± 0.3	125.6 ± 0.5	628
Methanol	2.8 ± 0.2	>400	>142.8
Ribavirin	10.4 ± 0.2	>400	>38.46

**Table 2.** Anti-Hepatitis C virus activities of *Acacia mangium* in single and combination with simeprevir.

Sample	50% inhibitory concentration *
*Acacia mangium* extract alone	4.75 ± 0.07 μg/mL
Simeprevir alone	19.65 ± 0.49 nM
	(0.0149 ± 0.0005 μg/mL)
Combination simeprevir and *Acacia mangium* extract	9.4 ± 0.2 nM
	(0.0069 ± 0.00014 μg/mL)

* Data represent the mean ± SD of three independent experiments

### Cytotoxic effects of *A. mangium* extracts

**Figure 2.**
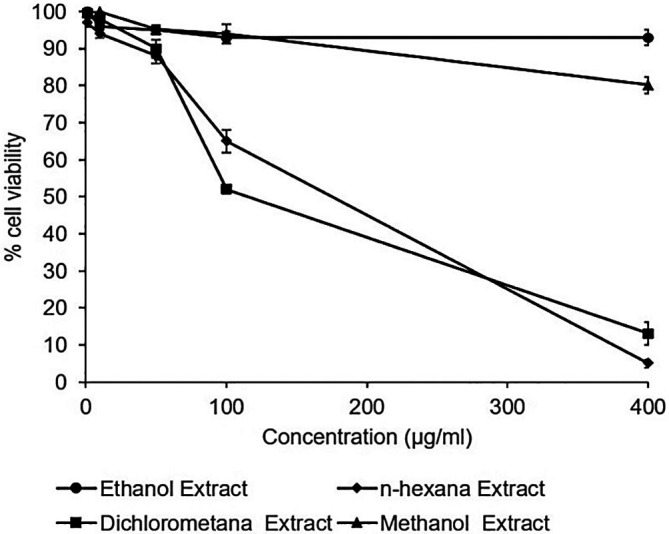
Percent viability of extracts in Huh7it-1 cells. Cells were treated with various concentrations of each extract of *Acacia mangium.* MTT (3-(4,5- Dimethylthiazol-2-yl)-2,5-Diphenyltetrazolium Bromide) reagent was added after 2 days of incubation, and cell viability was examined using a microplate reader at wavelengths of 450 and 630 nm. The percent cell viability was calculated, and the 50% cytotoxic concentrations were determined by SPSS probit analysis. The data represent the mean of three independent experiments.

The cytotoxicity assay of the extracts observed no toxic effect in the Huh7it. The percentage of cell viability demonstrated that all extracts possessed cell viability higher than 80% in the concentration of 400 μg/mL. However, n-hexane and dichloromethane extract showed a reduction in the percentage of cell viability at the dose of 400 μg/mL ( Figure 2). The raw data are available in the *Underlying data* ( Permanasari and Wahyuni, 2022c).

### Mode of action evaluation

**Figure 3.**
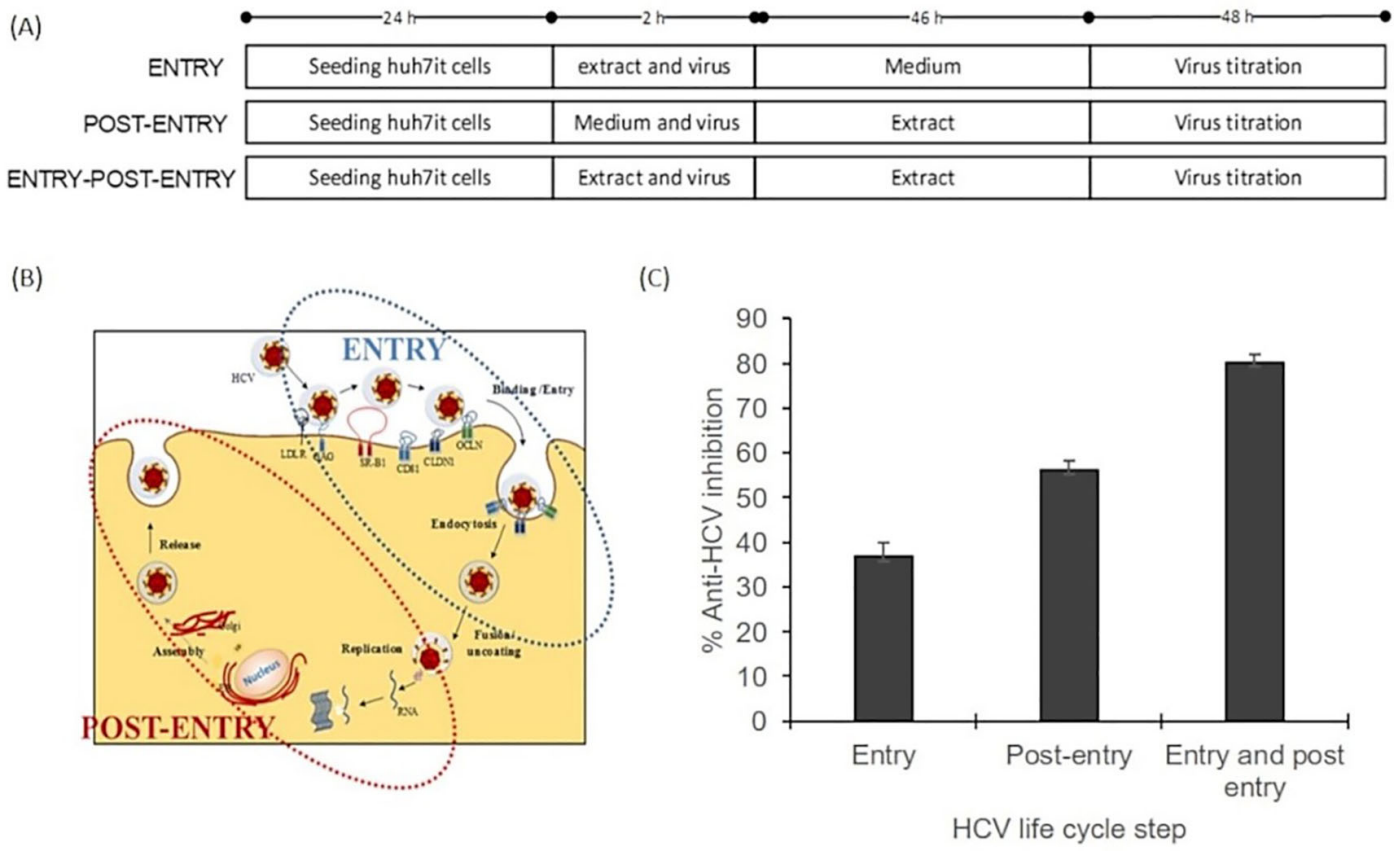
Mode of action analysis of the ethanol extract *of Acacia mangium* illustrated that HCV inhibition occurred dominantly in the post-entry step. (a) Cells were cultured with the ethanol extract of *A. mangium* (30 μg/mL) in three parallel experiments. First, cultured cells were treated with the extract only during inoculation (entry step). Second, cells were treated with the extract only after inoculation (post-entry step). Third, cells were treated with the extract both during and after inoculation. (b) The entry step comprises the processes of viral binding to host receptors, viral entry into the cells and endocytosis, whereas the post-entry comprises translation, replication, assembly, and release. (c) Percent inhibition of the entry step, post-entry step, and both steps. The data represent the mean ± SEM of three independent experiments

The mode of action assay was performed using three series of experiments. The result illustrated that the inhibitory effect was higher on the post-entry step than on the entry step. The percentage virus inhibition of post entry steps revealed more than 20% differences than the entry step ( Figure 3). This result suggested that the extract dominantly affected post-infection processes such as virus replication, virus assembly, and virus release. The raw data are available in the *Underlying data* ( Permanasari and Wahyuni, 2022d).

### Anti-HCV activity of the combination of *A. mangium* extract and simeprevir

Evaluation of the combination effect between the extract and simeprevir showed there is an enhancement of the effects of the extract to simeprevir, which is known as an HCV NS3 protein inhibitor. The addition of *A. mangium* extract increased the anti-HCV effect of simeprevir, as the IC _50_ of simeprevir when used in combination with *A. mangium* extract was reduced by 2-fold compared to that of simeprevir alone ( Table 2).

### Extract of *A. mangium* inhibited NS3 protein expression

**Figure 4.**
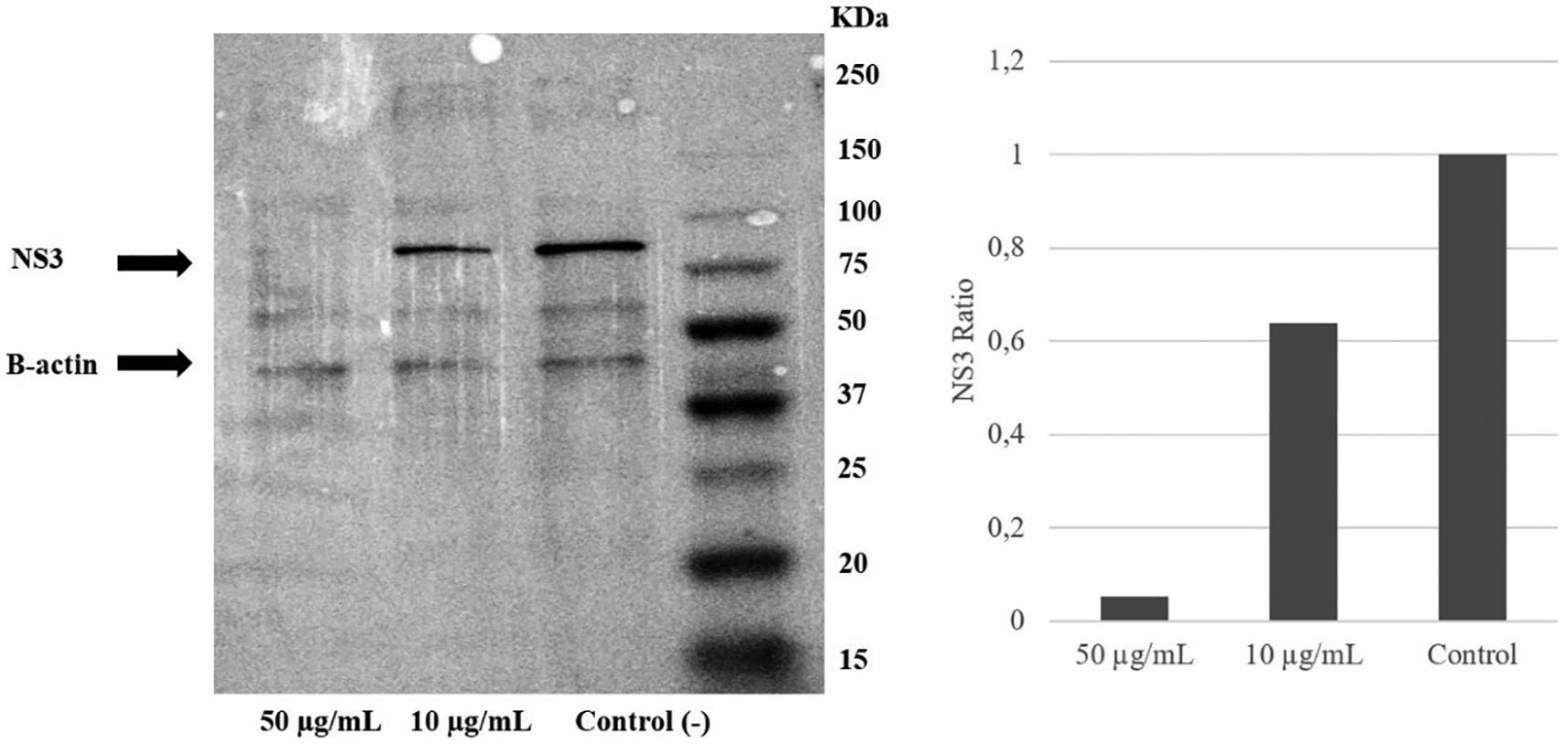
Extract of *Acacia mangium* reduced NS3 protein levels in a concentration-dependent manner. Huh7it-1 cells were treated with a mixture of the extract (final concentration, 10 or 50 μg/mL) and virus. The cells were lysed in RIFA buffer, and an equal amount of proteins were separated by SDS–polyacrylamide gel electrophoresis.

To examine the mechanism of action of the ethanol extract, western blotting analysis was performed. The result demonstrated a reduction of the NS3 protein level due to the extract intervention. Immunoblotting revealed that treatment with *A. mangium* extract at 10 or 50 μg/mL decreased the NS3 protein expression by 40% and 95%, respectively, versus the control ( Figure 4) ( *Underlying data*, ( Permanasari and Wahyuni, 2022e).

## Discussion

The study found that *A. mangium* possesses potential anti hepatitis C virus activity through some mechanism. The anti-HCV activities of the *A. mangium* leaves extracted using solvents of different polarities, namely 96% ethanol, n-hexane, dichloromethane, and methanol was examined. The data illustrated the strong anti-HCV activities of all extracts. Moreover, no toxic effect was observed according to the CC _50_ and selectivity index.

Ethanol is commonly used as a solvent in traditional drug development. Therefore, the strong anti-HCV activities of the ethanol extract and the lack of cytotoxicity provide necessary information for developing extracts of *A. mangium* as an alternative or complementary anti-HCV agent. However, we used various solvents with different polarities to further isolate the active anti-HCV compounds. The result demonstrated that the dichloromethane extract of *A. mangium* possessed the strongest inhibitory activity.

Mode of action analysis was performed as the first screening assay to determine the mechanism of *A. mangium* extract. The results illustrated that the ethanol extract more strongly inhibited HCV in the post-entry stage than in the entry stage. Virus production starts with binding of the virus to the host cell receptor, followed by virus entry and endocytosis. These processes are included in the entry step. The entry of HCV into hepatocytes is mediated by the viral E1 and E2 glycoproteins, which are the surface proteins of viral particles. HCV infection occurs through complex interactions of viral lipoviral particles with cellular factors, including low-density lipoprotein receptors, glycosaminoglycans, scavenger receptor class B type I, tetraspanin (CD81), claudin-1, and occludin ( Dubuisson *et al.*, 2008; Moriishi and Matsuura, 2003). Viral particles enter host cells through clathrin-mediated endocytosis, after which they are sent to the endosome. Meanwhile, the post-entry steps include translation, replication, and assembly. NS3–NS5 form a replication complex that produces new viral genomic RNA. Genomic RNA and HCV core proteins accumulate to form a nucleocapsid, which is excreted through the lumen side of the endoplasmic reticulum. After this excretion, the nucleocapsid can interact with very low-density lipoprotein (VLDL), followed by translocation to the Golgi for maturation. Mature HCV–VLDL complexes are released exocytically via the VLDL secretory pathway. Finally, new cells can be infected by released free HCV particles or by cell-to-cell transmission ( Fénéant *et al.*, 2014; Lindenbach and Rice, 2013; Zeisel *et al.*, 2015).

To further clarify the mechanism of the anti-HCV effects of *A. mangium* extract, Western blotting was performed to evaluate the effect of the extract on NS3 protein. *A. mangium* extract decreased NS3 protein levels versus the control. NS3 is a non-structural virus protein that plays an important role in replication. It is an attractive target for HCV treatment. Inhibition of NS3 could result in decreased virus production. Moreover, the anti-HCV activity of the extract was evaluated in combination with the NS3 protein inhibitor simeprevir. *A. mangium* extract was demonstrated to enhance the inhibitory activity of simeprevir against HCV. This suggested that the extract potentiated the effect of simeprevir on secondary targets of HCV.

**Figure 5.**
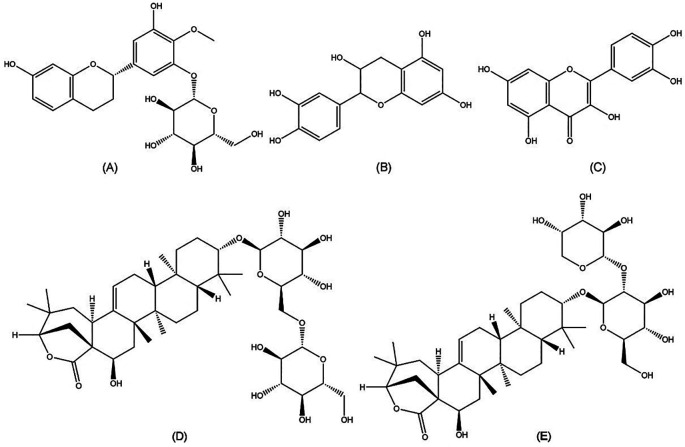
Molecular structures of five bioactive compounds from the *Acacia* genus. (a) Auriculoside ( https://pubchem.ncbi.nlm.nih.gov/compound/Auriculoside), (b) epicatechin ( https://pubchem.ncbi.nlm.nih.gov/compound/72276), (c) quercetin ( https://pubchem.ncbi.nlm.nih.gov/compound/5280343), (d) proacaciaside I ( https://pubchem.ncbi.nlm.nih.gov/compound/102446075), and (e) proacaciaside II ( https://pubchem.ncbi.nlm.nih.gov/compound/102446076).

Chemical compounds play an important role in anti-HCV activities. Further isolation of the active compounds from *A. mangium* against HCV is needed. However, it has been reported that *Acacia* species are rich in polyphenols, flavonoids, alkaloids, saponins, and terpenoids. It was reported compounds in the genus *Acacia* include epicatechin, quercetin, proacaciaside I, and proacaciaside II ( Figure 5). Those compounds were previously demonstrated to exhibit bioactivities such as anti-bacterial, anti-fungal, and anti-parasitic effects, which could contribute to anti-HCV properties ( Chew *et al.*, 2011; Rangra *et al.*, 2019).

## Conclusions

*A. mangium* leaf extracts possess strong anti-HCV activities without toxic effects. The extracts strongly inhibited the post-entry step, decreased NS3 protein levels, and enhanced the anti-HCV activities of simeprevir. These results suggest that *A. mangium* could be used to develop complementary and alternative treatments for HCV.

## Data Availability

Figshare: Plant Determination of *Acacia mangium.*jpg (It provided the taxonomy information of *Acacia mangium*) https://doi.org/10.6084/m9.figshare.20973511 ( Wahyuni, 2022) Figshare: Immunostaing HCV protocol. Immunostaining HCV.docx (It provided the detail steps of immunostainning process.) https://doi.org/10.6084/m9.figshare.20977168 ( Permanasari and Wahyuni, 2022a). Figshare: in vitro activity of *A. mangium* against HCV. raw data figure 1.docx (It demonstrated the figure of cells with infected cells, the tables which showed number of infected cells and percent inhibition of the three replication of experiments.). https://doi.org/10.6084/m9.figshare.20977714 ( Permanasari and Wahyuni, 2022b). Figshare: Cytotoxic effect of *A. mangium* extracts. cytotoxicity.docx (It provided the percent viability of extract ethanol, n-hexane, dichloromethane, and methanol https://doi.org/10.6084/m9.figshare.20977939 ( Permanasari and Wahyuni, 2022c) Figshare: Untitled Item. Mode of action.docx (It provided the number of infected cells in three kind of inoculation method,entry, post entry and both.). https://doi.org/10.6084/m9.figshare.20977933 ( Permanasari and Wahyuni, 2022d). Figshare: NS3 *Acacia mangium* inhibition. NS3 beta actin 4 (1).tif (It provided the raw data of western blotting assay) https://doi.org/10.6084/m9.figshare.21352095 ( Permanasari and Wahyuni, 2022e). Data are available under the terms of the Creative Commons Attribution 4.0 International license (CC-BY 4.0).
