# Supplementary material for: Acacia mangium: A promising plant for isolating anti-hepatitis C virus agents
Source: F1000Res. 2023 Aug 2;11:1452. Originally published 2022 Dec 8. [Version 3] doi: 10.12688/f1000research.124947.3 (PMC10690042; doi:10.12688/f1000research.124947.3)
Supplement: Peer review history

# Reviewer report: Reviewer response for version 3

**DOI:** 10.5256/f1000research.150283.r222958

**Published:** 2023-11-30

**Authors:** Moustafa Sarhan

**Affiliations:** 1 Zoology Department, Faculty of Science, Al-Azhar University, Assiut, Egypt

In the current study, the authors evaluated the anti-HCV activity of various extracts of *Acacia mangium* leaves. They also determined the cytotoxicity of the extracts. The mode of action experiments revealed the reduction of NS3 protein levels during the post-entry step with some effects on the entry step. Overall, the work is good however there are some concerns:

1. The title needs to be improved to be more indicative of the work, like this ...... "Evaluation of anti-hepatitis C virus of leaves extracts of *Acacia mangium"*
2. The graphs in Fig. 1 and Fig. 2 are not so clear. To make them more clear you can use colors for drawing the lines.
3. In the mode of action experiments, why you did not check the effect of the extract on the cells alone for 2 hours and then add the virus?
4. Why was ethanol extract selected to check the mode of action? Methanol extract also has a good effect! Dichloromethane has the highest effect and SI!
5. You may need to use fractionation-guided HPLC to find out the bioactive compound(s) in the leaf extract. You should add to the discussion and conclusion.

Is the work clearly and accurately presented and does it cite the current literature?

Partly

If applicable, is the statistical analysis and its interpretation appropriate?

Yes

Are all the source data underlying the results available to ensure full reproducibility?

Yes

Is the study design appropriate and is the work technically sound?

Yes

Are the conclusions drawn adequately supported by the results?

Partly

Are sufficient details of methods and analysis provided to allow replication by others?

Yes

Reviewer Expertise:

Molecular Biology, Antiviral peptides

I confirm that I have read this submission and believe that I have an appropriate level of expertise to confirm that it is of an acceptable scientific standard, however I have significant reservations, as outlined above.

# Reviewer report: Reviewer response for version 2

**DOI:** 10.5256/f1000research.144860.r172677

**Published:** 2023-06-05

**Authors:** Borris Rosnay T Galani

**Affiliations:** 1 Universite de Ngaoundere, Ngaoundere, Adamawa, Cameroon

In the submitted manuscript, Wahyuni *et al.* examined the *in vitro *antiviral effects of different organic extracts of *Acacia mangium* leaves on HCV infection in cell culture systems. The extracts' selectivity indices were determined using immunostaining and cytotoxicity tests. Western blot examination of the ethanol extract revealed that it primarily functions as a replication inhibitor, boosting simeprevir effectiveness when used in combination and significantly lowering NS3 viral protease expression compared to untreated controls. This research is original and contributes to our understanding of the biological potential of this plant in the treatment of viral hepatitis.

 However, certain flaws were discovered in this version that must be addressed before further consideration:

**Introduction:**

- Many references in the introduction are quite old, for example, Swain *et al.* 2010; Javed *et al.* 2011. The literature needs to be updated (2018-2023).
- New efforts also result in the approval of direct-acting antivirals, or DAAs, which aid in the recovery from HCV in three months. Sofosbuvir, Daclatasvir, and other DAAs are quite effective. The major restriction here is the emergence of resistance to these new medications. To validate the title of this article, I would recommend that the authors evaluate the antiviral effects of the plant extracts on HCV-resistant strains.
- Please write the species names in italics and correct spelling errors: *Acacia nilotica,* not 'Acacia nilotoca'.
- Simeprevir is an NS3/4A protease inhibitor used to treat HCV-genotypes 1 and 4 patients. Since the authors used this drug as a positive control in their tests, actual data on this compound needs to be presented in the introduction as well as its major limitation.
- Why did the authors confine the study to *in vitro* systems rather than including an in vivo examination? summaries of the opportunities provided by *in vitro* systems must be presented here.

**Methodology:**

- Please also include the length of the maceration of plant extracts.
- Please properly cite Wahyuni, 2013 and include it in the references. But this reference is also old.
- The Huh7it cell systems must be introduced correctly. What makes them superior to other JFH-1 systems?
- Two positive controls (ribavirin and simeprevir) were employed independently in this study with no evidence of their mode of action. To minimize confusion, this must be described explicitly. Also, clarify why Sofosbuvir a very popular replication inhibitor has not been used in this assessment. It would have helped to understand the extent of this effect on the replication step.
- For the suppression of HCV infectivity under the section "Anti-HCV activity," I would propose that the authors specify which viral proteins the primary (human) antibody employed for immunostaining precisely targets, and that they solely refer to prior publications (Permanasari & Wahyuni, 2022).
- I believe that at least one mean comparison test should be used to detect the statistical difference between IC50 values.
- What about the isobologram curves now that combination experiments have been completed? Before concluding the Simeprevir relationship, it would have been great to know whether this combination is synergistic or antagonistic.

**Results & Discussion**

- According to my interpretation of Table 1, the dichloromethane extract has the lowest IC50 value and the highest selectivity index (SI), and therefore should be used for further analysis. I'm curious why the authors chose to focus on the ethanol extract instead. Please stick to facts.
- Based on this result, I think the phytochemical characterization of the dichloromethane extract by HPLC/UV is necessary.
- Kindly support these findings with *in silico* studies of *Acacia mangium* compounds with the HCV NS3/4A protease.
- The information in Figure 1 and Table 1 is almost the same. I would advise the authors to concentrate solely on Table 1 because it includes IC50, CC50, and SI values, which are more relevant. Because IC50 is a result of a dose-dependent impact, showing it directly explains it and eliminates the need for Fig. 1.
- Fig 5: Honestly, I don't see the importance of these compounds since you have not isolated all these from your plant. In my opinion, you must isolate these compounds and test their effect on replication.
- What about the effects of these extracts on other non-structural proteins including the RdRp which is a key enzyme in the replication step?
- The effect of this extract on NS3/4A activity is also required

**Conclusion**

- Before concluding that *A. mangium *extracts might be exploited to produce supplementary and alternative therapies for HCVmore *in vivo* research is needed to evaluate the anti-HCV potential and assess the toxicity.

Is the work clearly and accurately presented and does it cite the current literature?

Partly

If applicable, is the statistical analysis and its interpretation appropriate?

Partly

Are all the source data underlying the results available to ensure full reproducibility?

Yes

Is the study design appropriate and is the work technically sound?

Yes

Are the conclusions drawn adequately supported by the results?

Yes

Are sufficient details of methods and analysis provided to allow replication by others?

Yes

Reviewer Expertise:

Pharmacology, Hepatology and Virology

I confirm that I have read this submission and believe that I have an appropriate level of expertise to confirm that it is of an acceptable scientific standard, however I have significant reservations, as outlined above.

# Response

**Published:** 2023-07-28

**Authors:** tutik sri wahyuni

**Affiliations:** Airlangga University, Indonesia

Dear Reviewer 

 Thank you for your comments and suggestions.

 Herewith our respond to the reviewer comments

 Introduction:

 Many references in the introduction are quite old, for example, Swain et al. 2010; Javed et al. 2011. The literature needs to be updated (2018-2023).

**Answer: **We revised some references.

 Javed was change to Segarra-Newnham M, See N, Fox-Seaman G. Retreatment of Hepatitis C Infection With Direct-Acting Antivirals. Fed Pract. 2020 Jul;37(7):316-319. PMID: 32908335; PMCID: PMC7473721.

 New efforts also result in the approval of direct-acting antivirals, or DAAs, which aid in the recovery from HCV in three months. Sofosbuvir, Daclatasvir, and other DAAs are quite effective. The major restriction here is the emergence of resistance to these new medications. To validate the title of this article, I would recommend that the authors evaluate the antiviral effects of the plant extracts on HCV-resistant strains.

**Answer:** Thanks for the recommendation. However, our main concern here is to show the potential of the *Acacia mangium* plant as a complementary drug against HCV. Therefore, we are also conducting combination treatment with simaprevir.

 Please write the species names in italics and correct spelling errors: Acacia nilotica, not 'Acacia nilotoca'.

**Answer:** The spelling and grammar errors have been proofread by a native English speaker and have been revised as per your suggestions.

 Simeprevir is an NS3/4A protease inhibitor used to treat HCV-genotypes 1 and 4 patients. Since the authors used this drug as a positive control in their tests, actual data on this compound needs to be presented in the introduction as well as its major limitation.

 Strengths of Simeprevir:

 High efficacy: Simeprevir has demonstrated high rates of sustained virologic response (SVR) in clinical trials, leading to viral clearance and improved patient outcomes.

 Genotype specificity: It is particularly effective against HCV genotypes 1 and 4, making it a valuable treatment option for patients infected with these genotypes.

 Once-daily dosing: Simeprevir is typically administered as a once-daily oral medication, which improves convenience and patient adherence to the treatment regimen.

 Weaknesses of Simeprevir:

 Resistance development: The long-term use of Simeprevir, especially as a monotherapy, can lead to the development of viral resistance, potentially reducing its effectiveness.

 Limited efficacy in certain populations: Simeprevir may not be as effective in certain patient populations, such as those with liver cirrhosis or prior treatment failure.

 Drug interactions: Simeprevir has the potential for drug interactions, particularly with certain medications metabolized by cytochrome P450 enzymes, which may require dosage adjustments or careful monitoring.

 Reference:

 Rice CM, et al. (2014). "Simeprevir - a protease inhibitor for the treatment of hepatitis C virus infection." N Engl J Med, 370(8): 796-797.

 Why did the authors confine the study to in vitro systems rather than including an in vivo examination? summaries of the opportunities provided by in vitro systems must be presented here.

**Answer: **The study of in vitro systems refers to conducting experiments or investigations in a controlled laboratory environment outside of a living organism. In vitro studies involve using cells, tissues, or organs cultured in a laboratory setting, often in artificial conditions that mimic aspects of the natural environment. And for HCV the in vivo model was very limited

 Methodology:

 Please also include the length of the maceration of plant extracts.

**Answer:** The maceration process has been described

 Please properly cite Wahyuni, 2013 and include it in the references. But this reference is also old.

**Answer**: The appropriate method was described very well in Wahyuni 2013. And the method was used the same until now.

 The Huh7it cell systems must be introduced correctly. What makes them superior to other JFH-1 systems?

**Answer:** The Huh7 cell system, is a widely used in vitro cell culture model for studying the hepatitis C virus (HCV). It is derived from the Huh-7 human hepatoma cell line and has been extensively employed for HCV research, including the study of the JFH-1 strain of the virus.

 Two positive controls (ribavirin and simeprevir) were employed independently in this study with no evidence of their mode of action. To minimize confusion, this must be described explicitly. Also, clarify why Sofosbuvir a very popular replication inhibitor has not been used in this assessment. It would have helped to understand the extent of this effect on the replication step.

**Answer:** We used ribavirin and simeprevir that still used as anti-hcv drug in some area in developing countries due to limited others DAAs

 For the suppression of HCV infectivity under the section "Anti-HCV activity," I would propose that the authors specify which viral proteins the primary (human) antibody employed for immunostaining precisely targets, and that they solely refer to prior publications (Permanasari & Wahyuni, 2022).

**Answer: **We used the serum of a patient with a positive HCV genotype 2a, which did not have a specific viral protein target.

 I believe that at least one mean comparison test should be used to detect the statistical difference between IC _50_ values.

**Answer: **We complete it with Student's t-test: The t-test is a parametric statistical test that compares the means of two groups. Since we have two sets of IC _50 _values

 What about the isobologram curves now that combination experiments have been completed? Before concluding the Simeprevir relationship, it would have been great to know whether this combination is synergistic or antagonistic.

**Answer:** We have completed with the diagram which showed synergetic effect by Combination Index (CI) analysis through compusyn software. The Combination Index is a quantitative measure used to evaluate drug interactions in combination therapies. It is calculated based on the dose-response curves of individual drugs and the combination. The CI value assesses whether the observed effect of the combination is synergistic (CI < 1), additive (CI = 1), or antagonistic (CI > 1).

 Results & Discussion

 According to my interpretation of Table 1, the dichloromethane extract has the lowest IC50 value and the highest selectivity index (SI), and therefore should be used for further analysis. I'm curious why the authors chose to focus on the ethanol extract instead. Please stick to facts.

**Answer:** The author was more explored in ethanol extract because of the potency in developing extanol extract as herbal drug product.

 Based on this result, I think the phytochemical characterization of the dichloromethane extract by HPLC/UV is necessary.

 Kindly support these findings with in silico studies of Acacia mangium compounds with the HCV NS3/4A protease.

**Answer:** We will conduct it on the future experiments

 The information in Figure 1 and Table 1 is almost the same. I would advise the authors to concentrate solely on Table 1 because it includes IC50, CC50, and SI values, which are more relevant. Because IC50 is a result of a dose-dependent impact, showing it directly explains it and eliminates the need for Fig. 1.

**Answer: **The figure 1 we described the doses dependent manner among concentration and percent inhibition.

 While in the table represent the IC50, CC50 and SI, so we think it also necessary to include figure 1.

 Fig 5: Honestly, I don't see the importance of these compounds since you have not isolated all these from your plant. In my opinion, you must isolate these compounds and test their effect on replication.

**Answer:** The five compounds were active compounds that showed potential as both antibacterial and antiviral agents since we did not perform the isolation. This information will be very useful for predicting compounds that may play as anti-HCV agents. We agree that more study was needed to elucidate the compounds and determine their activities.

 What about the effects of these extracts on other non-structural proteins including the RdRp which is a key enzyme in the replication step?

**Answer:** We not perform the PCR for RdRp. Our study was detected the NS3 protein level, that also a key component of the hepatitis C virus (HCV) replication. It plays essential roles in viral replication and is considered an attractive target for antiviral drug development.

 The effect of this extract on NS3/4A activity is also required

 We agree with the reviewer suggestion, however some limiting of experiment condition that could not examine that matter

 Conclusion

 Before concluding that *A. mangium* extracts might be exploited to produce supplementary and alternative therapies for HCV more in vivo research is needed to evaluate the anti-HCV potential and assess the toxicity.

**Answer:** Yes, sure. However, our concern was to analyze the activity of Acacia mangium in inhibiting HCV growth in an in vitro model.

 The in vivo study will be conducted the the future research.

# Reviewer report: Reviewer response for version 2

**DOI:** 10.5256/f1000research.144860.r170379

**Published:** 2023-05-19

**Authors:** Courage Sedem Dzah

**Affiliations:** 1 Ho Technical University, Ho, Volta Region, Ghana

The authors have studied the antiHCV effect of leaf extracts of *Acacia mangium*. It is a good work as natural bioactives are being exploited for beneficial use. Value addition is important and so, I think this work is very good in that sense. However, there are a few issues to be addressed technically:

**Abstract**

1. "... *Acacia  mangium *is one of the *Acacia *genus that contain with various metabolites..." - Take out the "with" I have underlined.
2. "The current study examined the anti-hepatitis C virus (HCV) activities of *Acacia mangium* extracts in solvents with various polarities and further evaluated the mechanism of action of the extracts on t he protein virus and combination treatment models." - Are you evaluating effects on combination treatment models too or on protein virus? I do not think the models are also dependent variables. 
3. "Anti-hepatitis C virus activities was conducted by *in vitro* culture cells..." - Please replace "by" with "using". Because culture cells cannot conduct a test. Also, better to say cell culture than "culture cell".
4. "Further examined its NS3 protein inhibition was evaluated by western blotting assay..." - This whole statement is grammatically incorrect. Take out "examined" and replace "by" with "using".
5. What is ribavirin? Any brief details on it and why was it used as a control?
6. "The mode of action of the ethanol extract was evaluated at 30 µg/mL, revealing that the inhibitory effect was stronger on the post-entry step than on the entry step." -  what does entry and post-entry steps mean in this context and why study kinetics at a higher concentration than the 4.6 ug/ml for ethanol extract?
7. Why use ribavirin as a positive control and also use simeprevir in combination treatment? Comparing simeprevir-based treatment with the control will not make sense. 
8. "...The current study examined the anti-hepatitis C virus (HCV) activities of *Acacia mangium* extracts in solvents with various polarities..." - Please indicate here early enough that you used the leaves of the plant. This was not mentioned until your conclusion and it should not be so. 

**Methods**

1. Why extract compounds from 200g of leaf powder? What informed this figure? 
2. At what temperature was the extraction done?
3. For how long was the maceration done?
4. For your MTT assay, why did you not rinse your cells and take out media and add DMSO before incubating again for colour development for spectral reading? DMSO is added to MTT assays at the end of the experiment to dissolve the formazan crystals that were created during the experiment. If DMSO is not added and the formazan crystals are not dissolved, this would cause significant changes in the absorbance spectrum due to the sodium bicarbonate that would still be there. 

**Cytotoxic effect result**

1. How sure are you that you have removed interference from cell culture media and that your result is not as a result of potential interference?

**Other concerns**

1. What compounds from the plant leaf specifically are responsible for the results you have gotten? 
2. Figure 5 throws light on 5 bioactive compounds. Are the observations as a result of all of these compounds or some of them or just one of them?
3. Does it mean that the results are only due to these five compounds or there may be other compounds beyond these five that may give the results you have?
4. Please, do consider English Language Editing to avoid some errors. 

Thank you for the opportunity.

Is the work clearly and accurately presented and does it cite the current literature?

Partly

If applicable, is the statistical analysis and its interpretation appropriate?

Yes

Are all the source data underlying the results available to ensure full reproducibility?

Partly

Is the study design appropriate and is the work technically sound?

Partly

Are the conclusions drawn adequately supported by the results?

Partly

Are sufficient details of methods and analysis provided to allow replication by others?

Yes

Reviewer Expertise:

1. Plant bioactive compounds extraction, purification, identification, characterization, bioactivity assessment, bioavailability and potential industrial applications (e.g. bioactive compound-based nanoparticles, nano-emulsions, etc)  2. Food Science and Engineering. 3. Physical processing and treatment of biomass

I confirm that I have read this submission and believe that I have an appropriate level of expertise to state that I do not consider it to be of an acceptable scientific standard, for reasons outlined above.

# Response

**Published:** 2023-06-01

**Authors:** tutik sri wahyuni

**Affiliations:** Airlangga University, Indonesia

Dear Reviewer, 

 Thank you for your comment and suggestion. 

 Here with some point of our responses: 

 1. "... *Acacia mangium *is one of the *Acacia *genus that contain with various metabolites..." - Take out the "with" I have underlined.

**Answer**: We have revised the manuscript and remove the word ”with”

 2. "The current study examined the anti-hepatitis C virus (HCV) activities of *Acacia mangium* extracts in solvents with various polarities and further evaluated the mechanism of action of the extracts on t he protein virus and combination treatment models." - Are you evaluating effects on combination treatment models too or on protein virus? I do not think the models are also dependent variables. 

**Answer**: Our study evaluated the effect of extract in the NS3 protein of virus. And it demonstrated the inhibition effect of extract in NS3 protein level of virus. Furthermore, the combination analysis was conducted between extract and simeprevir which showed enhancement effects of extract on anti-HCV activity of simeprevir. Those results suggested that extract has a role in protein inhibition.

 However, our study did not examine directly the mixture of extract and simeprevir on protein virus.

 3. "Anti-hepatitis C virus activities was conducted by *in vitro* culture cells..." - Please replace "by" with "using". Because culture cells cannot conduct a test. Also, better to say cell culture than "culture cell".

**Answer**: Thank you for the suggestion. We have revised in the manuscript, changed the word 'by' with 'using' and changed 'culture cells' with 'cell culture'. 

 4."Further examined its NS3 protein inhibition was evaluated by western blotting assay..." - This whole statement is grammatically incorrect. Take out "examined" and replace "by" with "using".

**Answer**: We have revised in the manuscript

 5. What is ribavirin? Any brief details on it and why was it used as a control?

  Answer: Ribavirin was used as a treatment in hepatitis C virus infection. It has several mechanism, (1) Modulate the host immunity by inferfare the T helper 1 and T helper 2, (2) Inhibit the inosine monophosphate dehydrogenase and depletion of guanosine triphosphate which resulting the inhibition of viral replication and protein synthesis, (3) ribavirin also act as mutagen in the viral target.

 Although the Direct Acting Antiviral Agents have been used as current treatment of hepatitis C virus, however some developing area still used ribavirin and specific patient condition was need with ribavirin combination treatment.

 6. "The mode of action of the ethanol extract was evaluated at 30 µg/mL, revealing that the inhibitory effect was stronger on the post-entry step than on the entry step." - what does entry and post-entry steps mean in this context and why study kinetics at a higher concentration than the 4.6 ug/ml for ethanol extract?

**Answer**: The Hepatitis C Virus life cycle is mainly divided into two stage; entry step (viral adsorption and penetration) and post-entry step (uncoating, translation, replication, assembly and release). The time-of-addition study is performed to determine antiviral effect of the plant extract in the HCV life cycle, and the plant extract concentrations which exhibits >90% virus inhibition (30 µg/mL in this experiment) is usually used to analyse.

 7. Why use ribavirin as a positive control and also use simeprevir in combination treatment? Comparing simeprevir-based treatment with the control will not make sense. 

**Answer**: The purpose using ribavirin as positive control not for comparing with simeprevir. We use simeprevir as a combiantion treatment due to the mechanism of simeprevir as NS3 protein inhibitor.

  8. "...The current study examined the anti-hepatitis C virus (HCV) activities of *Acacia mangium* extracts in solvents with various polarities..." - Please indicate here early enough that you used the leaves of the plant. This was not mentioned until your conclusion and it should not be so. 

**Answer**: We have revised by added leave in the introduction section. We have mentioned in the material section that leaves were used of *Acacia mangium* extract.

 9. Why extract compounds from 200g of leaf powder? What informed this figure? 

**Answer**: Plant material preparation was derived from the powder of dried leaves. A number of 200 g was used for extraction process with ethanol solvent and other 200 g of *A. mangium* powder was extracted with difference polarities solvent, n-hexane, dichloromethane and methanol). 200 g of sample is enough for the research purpose.

 10. At what temperature was the extraction done?

**Answer**: The extraction was done in the room temperature.

 11. For how long was the maceration done?

**Answer**: The maceration was done overnight in three times.

 12. For your MTT assay, why did you not rinse your cells and take out media and add DMSO before incubating again for colour development for spectral reading? DMSO is added to MTT assays at the end of the experiment to dissolve the formazan crystals that were created during the experiment. If DMSO is not added and the formazan crystals are not dissolved, this would cause significant changes in the absorbance spectrum due to the sodium bicarbonate that would still be there. 

**Answer**: The MTT assay was conducted as you was mentioned. We have revised in the manuscript.

 After 2 days incubation, the medium was discarded, rinse with medium then 150 mikroliter of 10% of MTT reagent was added and incubate for 4 hours. After incubation time removed the MTT and DMSO was added to dissolve the formazon.

 13. How sure are you that you have removed interference from cell culture media and that your result is not as a result of potential interference?

**Answer**: The cytotoxic assay was done following our previous method and replication was also conducted to ensure the valid result.

 Other concerns

 14. What compounds from the plant leaf specifically are responsible for the results you have gotten?

**Answer**: Our study have not deermine the single compounds with responsible to the activities. Following to the references epicatechin, quercetin, proacaciaside I, and proacaciaside II were reported as isolated compounds of Acacia genus that may contribute in their activities.

  15. Figure 5 throws light on 5 bioactive compounds. Are the observations as a result of all of these compounds or some of them or just one of them?

**Answer**: The compounds in Figure 5 were known as isolated compounds of Acacia genus that may give contribution in its activities.

 16. Does it mean that the results are only due to these five compounds or there may be other compounds beyond these five that may give the results you have?

**Answer**: Our studies have not determined the specific compounds with contribute for its anti-HCV activities. There are strill many unidentified compounds from this plants.

 17. Please, do consider English Language Editing to avoid some errors. 

**Answer**: We have done the proof read editing and revised some portion.

 Thank you

# Reviewer report: Reviewer response for version 1

**DOI:** 10.5256/f1000research.137197.r162586

**Published:** 2023-02-21

**Authors:** Ziwen Wang

**Affiliations:** 1 Tianjin Normal University, Tianjin, China

Current study evaluated the anti-HCV activity of various extracts of *A. mangium*. In addition, the cytotoxicity of the extracts was evaluated. The mode of action was additionally assayed to determine the part(s) of the HCV life cycle inhibited by the extracts. Moreover, its mechanism of action was examined by Western blotting and combination treatment with current anti-HCV drug. The work is important for the development of novel antivirals, and the results are interesting. I recommended it to be indexed after minor revision.

 For Abstract: Parts Background and Methods need to be reorganized, and the content does not match the requirements. Part Background only needs to introduce the research background. Part Methods does not need to write the specific operation process.

 For Introduction: The research progress on the biological activity of *Acacia mangium* extract should be increased.

 Table 1: Extracts from different solvents show different activities because the chemical components in them have changed. Can you detect which components have changed by liquid chromatography? In addition, have you ever tested the biological activity of different batches of extracts from the same solvent?

Is the work clearly and accurately presented and does it cite the current literature?

Yes

If applicable, is the statistical analysis and its interpretation appropriate?

Yes

Are all the source data underlying the results available to ensure full reproducibility?

Partly

Is the study design appropriate and is the work technically sound?

Yes

Are the conclusions drawn adequately supported by the results?

Yes

Are sufficient details of methods and analysis provided to allow replication by others?

Yes

Reviewer Expertise:

Discovery of novel antivirals based on natural products

I confirm that I have read this submission and believe that I have an appropriate level of expertise to confirm that it is of an acceptable scientific standard.

# Response

**Published:** 2023-02-24

**Authors:** tutik sri wahyuni

**Affiliations:** Airlangga University, Indonesia

Dear Reviewer, 

 Thank you for the review comments. 

 Herewith I submitted the revised version of manuscript following the reviewer comments. 

 We revised the abstract section in the part of introduction and method. Other revision was also mentioning the bioactivities information of *Acacia mangium* and other Acacia genus in the introduction section. 

 Other comments were regarding the HPLC profile of each extracts that have differences polarities. Not much information regarding this data, however some information of *A. mangium* compounds and other *Acacia *genus have been explained in the text. 

 Comment regarding the examination of difference batch of extract have been done and gave the relatively the same activity. 

 Best regards,

 Tutik Sri wahyuni, PhD.
